# The Administration of Anæsthetics

**Published:** 1870-08

**Authors:** A. Ernest Sansom

**Affiliations:** Physician to the Royal Hospital for diseases of the Chest, &c. &c.


### ARTICLE VI.

### *The Administration of Anæsthetics.*

By A. Ernest Sansom, M. D.,

Physician to the Royal Hospital for diseases of the Chest, &c. &c.

The public as well as the professional mind seems at the present time to be passing through one of those phases of terrorism which, induced by the news of a death during the administration of chloroform, occur from time to time. It is vain to adduce the argument that, considering the vast number of times in which an anæsthetic is administered with comfort and safety, the danger is, proportionately, exceedingly small. If but one in ten thousand die, it is right that one death awaken all our powers to avert the danger from those who come after.

It is a right time that we should take a retrospective glance, and attain an estimate of the present position of anæsthetic agents and their administration.

In the first place, we are not, as formerly, confined to the use of one agent. To ether succeeded chloroform, to chloroform amylene, tetrachloride of carbon, bichloride of methylene, nitrous oxide, besides many other agents less widely known, and various mixtures and superadditions of one anæsthetic and another. Chloroform, the most widely employed, takes almost all the blame of the disasters. To my mind, we are too much disposed to look upon the production of a state of insensibility, for the performance of a surgical operation, as the administration of an agent rather than the induction of a state or condition. It is not the administration of chloroform, but the induction of anæsthesia which is required. We have a power of selection now, which was not possessed in times past; a fatal case points less to the failure of an agent than to the imperfection of a system.

The first rule for guidance is—

*A*. The choice of an anæsthetic agent should be regulated by the condition of the patient and the requirements of the case.

If it be desired to relieve pain, as in headache or the slighter forms of neuralgia, I know no better agent than tetrachloride of carbon, whatever be the condition of the patient. If an operation of short duration is to be performed, the administration of nitrous oxide is probably the most indicated. This has the properties of quick action, induction of deep insensibility with rapid recovery. The use of this agent requires, however, apparatus and time for arrangement; it cannot be a measure for the many and for the moment. Yet, largely used as it is by dentists, it takes away from the general dangers of anæsthesia, by withdrawing from the greater risks of the other anæsthetics, a large class which was proved to be in aforetime peculiarly prone to danger. Certain conditions should, however, in my opinion, preclude the administration of nitrous oxide; these are pulmonary congestion, cardiac affections, and cerebral affections. When an operation of uncertain or of long duration is to be performed, the chief agents are ether, bichloride of methylene, and chloroform. Our data as to the bichloride of methylene are, I think, as yet insufficient. As we have seen lately, one death has resulted from its use. In a case in which I administered it for a dental operation, signs of syncope occurred and I was obliged to substitute chloroform. The great question is does bichloride of methylene sustain the heart's action during profound anæsthesia with greater power than chloroform? To this question a wide practical experience (which, from *a priori* evidence, the agent greatly deserves) only can reply.

The agents which remain to be considered are ether and chloroform. Concerning the comparitive action of these, I think we are justified in concluding that, while the latter is by far the more practicable agent, ether can induce a profouud anæsthesia with less depression of the heart's action than chloroform. If complete muscular relaxation be required, as for the reduction of dislocation, etc., it is better to use ether.

For the great majority of cases the chief agent remains chloroform. The contra-indications to its use are the existence of prominent signs of fatty degeneration of the heart (valvular disease is no contra-indication), *delirium tremens*, and habitual intemperance. In the first of these conditions it will be a question whether any anæsthetic at all shall be administered. Argument may fairly urge that the shock of an operation will be a danger to the feeble heart. On the other hand, there is the undoubted fact that chloroform can induce cardiac debility. The dictum that " a case for operation is a case for chloroform, " can be at once set aside as a finality which would arrest all one's efforts to impart greater safety into the practice of anæsthesia. Fortunately we have a method of relieving which can, as M. Claude Bernard has shown, go hand in hand with the operation of a volatile anæsthetic—the subcutaneous injection of morphia. Supposing, therefore, a patient having undoubted signs of cardiac degeneration be the necessary subject of an operation, I think no better plan could be counselled than the subcutaneous injection of morphia, followed, if necessary, by the inhalation of ether. In cases where the patient is the subject of habitual intemperance, the subcutaneous injection of morphia also is indicated. With this preparation chloroform may with care be administered instead of ether, which has a longer stage of excitement. Having made choice of an anæsthetic, another rule presents itself to us.

*B.* Especial care should be taken at the early stages of the administration of an anæsthetic, whatever that anæsthetic may be.

There is no doubt that the emotional disturbances which occur at such a time may induce dangerous and even fatal effects. Sudden death at the moment of commencement of an operation when no anæsthetic whatever has been employed, are far from uncommon. Such instances have occurred in the practice of Stanley, Desault, Civiale, Roux, Chopart, Cazenave, Gargangeot, and others, and have been recorded by many observers. Giraldes goes so far as to say that " the accidents which have been observed do not much surpass in number the cases of sudden death which have occurred without apparent cause during or soon after operations." From collateral evidence, however, we know that this though a partial, is not a complete explanation of chloroform dangers. It is a significant fact that nearly half of the cases which have proved fatal (50 in 190) succumbed before the full effect of chloroform was induced. This leads to the consideration of a word, which I confess I do not like, that has been adduced to explain the catastrophies—idiosyncrasy. What does it mean? A condition of system inimical to chloroform *qua* chloroform? It cannot be so, for we see that emotional syncope may occur without the anæsthetic element. Just as some people faint at slight causes—the scratch of a finger, the sight of blood, or an emotional effort—so some fatally faint at the commencement of an operation. Moreover, some of those who have suddenly died in the early stages of chloroform administration have previously inhaled with impunity. It follows that every effort should be made that the emotional disturbances should be calmned, and that the early stages of an administration should be conducted with care and caution. This eliminates one of the elements of the so-called idiosyncrasy inimical to chloroform; it is a condition of sympathetic susceptibility induced by any external agency. It will not, however, explain those deaths which occur in the more profound stages. we are led to a third rule—

*C.* In the administration of chloroform due regard should be paid to its well established physiological properties. This may almost seem a truism. Yet I think that nearly all who have made this subject a special study will agree with me that the toxic action of chloroform is ignored in the common method of administration. No one can from experiment or from observation of the records doubt for a moment that chloroform does not possess a toxic action; and just as with all other agents its danger is dependent upon its dose. The most competent authorities agree that too strong atmospheres of chloroform are dangerous. For the purpose of inducing and maintaining anæsthesia a portion of no more than four per cent. of chloroform vapour in atmospheric air is necessary; above six per cent. is dangerous. Strong atmospheres of chloroform administered to the human subject are dangerous in two ways—First, by their tendency, acting on the branches of the *parvagum* distributed to the lung, and thus by reflex action upon the heart, to cause shock or cardiac syncope.* This may occur at the early stages of anæsthesia

* On this point see an exhaustive review of the whole subject of anæsthesia in the *American Journal of the Medical Sciences* for January, 1867, and Brown Sequard's Lectures on the Physiology and Pathology of the Nervous system, p. 160, or any period prior to complete loss of consciousness. secondly, by direct paralysis of the cardiac and vaso-motor nerves. This can occur only in profound anæsthesia, though it also may be rapidly induced. When pure chloroform is sprinkled upon a piece of lint or a napkin, what percentage of the vapour is it possible for a patient to inspire? I have been at some pains to investigate this, and it may cause some surprise to those who have not practically inquired into the point to be assured that it is possible for a patient at a temperature of from 60° to 64° Fahr., when a drachm of chloroform is sprinkled upon lint, to inspire an atmosphere containing more than thirteen per cent. of the vapour.†

Of course a happy chance so regulates the matter that a patient does not usually inhale this proportion, and a skilled administrator will take care that he does not; but the possibility of the danger remains. How often in the details of the fatal cases it is recorded in some such fashion as this, "More chloroform was poured upon the lint, the patient, inhaled and fell back, etc. etc." Why? because he suddenly inhaled a deadly dose.

Granted that a due dilution of chloroform is a necessary thing, how shall we obtain it? There is a widely spread objection against inhalers; they are not at hand at the moment they are wanted. I think they can be dispensed with if the chloroform employed be diluted with an equal bulk of absolute alcohol. By such a measure as this I have ascertained that, partly by the admixture of the chloroform vapour with the vapour of alcohol, but chiefly by the retarding power which the latter exerts upon the volatilization of the chloroform, the percentage in the resultant atmosphere is reduced just one half. The above consideration eliminates another element so-called idiosyncrasy. The effect of a sudden inspiration of a strong chloroform atmosphere is just as much toxic as the swallowing of an overdose of prussic

† See tables appended to my paper read at the British Medical Association at Oxford, and reprinted from *British Journal of Dental Science* for September, 1868, and "On the Parturition and Anæsthetics in Obstetric Practice" in *Transactions* of Obstetrical Society of London, vol. x. p. 161.

acid. But it may now be urged that in some cases wherein you have exercised all care, and while you are administering only a proper dose of chloroform vapour, from some cause (perhaps connected with the operation) the heart may show sign of failing. We are led to what I think may be established as a rule.

*D.* In the course of the induction or during the maintenance of anæsthesia one agent may be advantageously substituted for another. Practically, in animals, as well as in man I have found that if in the course of chloroform administration there has been the least sign of circulatory debility ether substituted for the chloroform renews the power whilst maintaining the anæsthesia.‡ This is confirmed by my friend Mr. Geo. Cowel, in a letter to these columns. In observations upon animals I have found that the substitution of any one anæsthetic for any other stimulates the heart.

*Practical Rules.*—Selection of cases having been made according to the principles before mentioned, I would suggest the following as rules for guidance:—

1. The administrator intrusted with the induction of anæsthesia should be provided with the following armamentarium: A case of three stoppered bottles, one containing the mixture of chloroform and absolute alcohol, another pure chloroform a third absolute ether. A soft hankerchief or porous material through the fibres of which, when covering the face, respiration can readily take place. A hypodermic syringe with suitable morphia solution.

2 The patient should have abstained from food as far as possible on the day of inhalation. Nothing whatever should have been taken for hours.

3. The patient being, if possible, in the recumbent position, the administrator should place the hankerchief over the nose and mouth, so as loosely to cover them with a single fold, and note that respiration is unimpeded. The mixture of chloroform and alcohol should then be dropped on the fold, a few minims at first, and, after two or three inspirations,

‡ See Observations by Dr. Greenhalgh, *Obst. Trans.*, vol. viii. p. 53.

twenty or thirty, which should be renewed as often as evaporation renders necessary. This should be continued until the desired stage of anæsthesia is arrived at.

4. Ether may now be substituted if it is thought desirable, the fold of lint being moistend therewith.

5. In some rare cases it may be necessary (but only after tolerance has been induced by the administration of the dilution) to use some of the pure chloroform.

6. In case of any alarming symptoms, artificial respiration should immediately be practised.—*London Med. Times and Gazette.*

---

